# New Appliances and Things Medical

**Published:** 1908-11-28

**Authors:** 


### NEW APPLIANCES AND THINGS MEDICAL.

[We shall be glad to receive at our Office, 28 & 29 Southampton Street, Strand, London, W.C., from the manufacturers, specimens of all new preparations and appliances which may be brought out from time to time.]

### A SANITARY SOAP CONTAINER.

The use of ordinary cake soap for cleansing the hands in operating theatres, out-patient rooms, and hospital wards, although still very common, has several obvious disadvantages. In modern theatres the hot and cold water taps are worked by the foot or knee, and the nail brushes are boiled frequently and kept in carbolic; but to render hand-contamination still less likely during the process of washing, some form of liquid soap in an efficient container is clearly desirable.

The specimen which we have received of the West Disinfecting Company's Liquid Soap Container seems to fulfil all the requirements of the case, and after investigation we can recommend it to the notice of medical practitioners and hospital authorities. It consists of a strong glass globe suspended in a metal frame, whose base is securely weighted. The globe contains liquid soap, which is discharged through a small nozzle, a few drops at a time, whenever the globe is rotated through a quarter of a circle. The act of tilting is effected by light downward pressure on a projecting metal plate, and, directly this pressure is withdrawn, the globe swings back by its own weight to the former position. The metal fittings are simple and durable, and there is nothing in the apparatus to get out of order. By its use each person receives a fresh supply of soap sufficient for his immediate needs, and the remaining soap is protected from all contamination. The container is thus both cleanly and economical. The liquid soap supplied is prepared from vegetable oils, and without excess of alkali and it is therefore not irritating to the hands, while a good lather can be obtained with it. One filling is said to be sufficient for from 500 to 700 washings, and one gallon of soap for nearly 1,000 washings. There are of course many other places, besides hospitals and surgeries, in which the use of this apparatus would be beneficial; and it is possible that in the course of time the educated public may begin to look with disfavour on common cakes of soap in lavatories and hotels.

The sole agent for this country is Charles Flint, 25 Victoria Street, London, S.W. The cost of the soap dispenser is 10s. 6d., or less if supplied on lease. The liquid soap costs from 10s. 6d. a gallon.

